# Supplementary material for: A mouse model that is immunologically tolerant to reporter and modifier proteins
Source: Commun Biol. 2020 May 29;3:273. doi: 10.1038/s42003-020-0979-0 (PMC7260180; doi:10.1038/s42003-020-0979-0)
Supplement: Supplementary file 1 — Supplementary information [file 42003_2020_979_MOESM1_ESM.pdf]

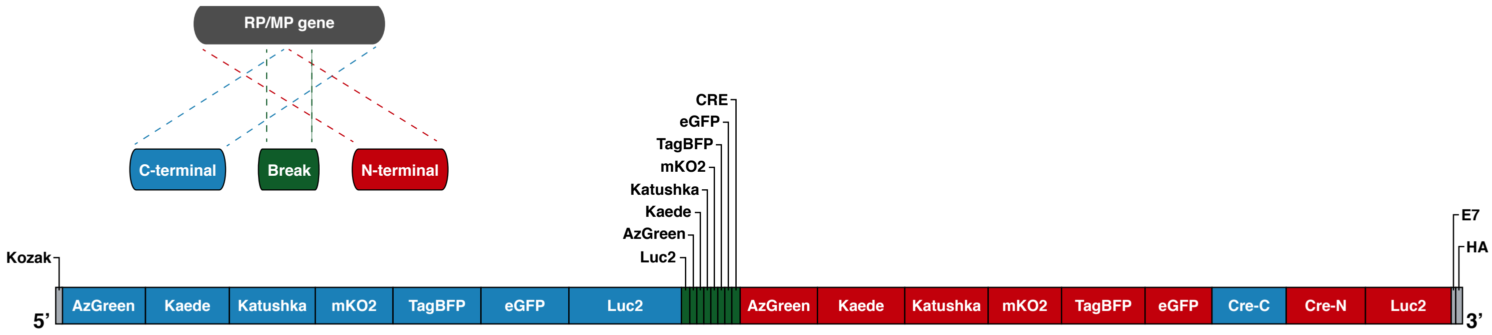


**Supplementary Fig. 1. Design of the *Tol* open reading frame.** Cartoon depiction of the *Tol* open reading frame (ORF), with the position of each gene fragment depicted. AzGreen = Azami Green, mKO2 = mKusabira Orange2, TagBFP = TagBlue fluorescent protein, eGFP = enhanced green fluorescent protein, Luc2 = luciferase2, E7 = HPV $E7_{49\text{-}57}$ epitope.

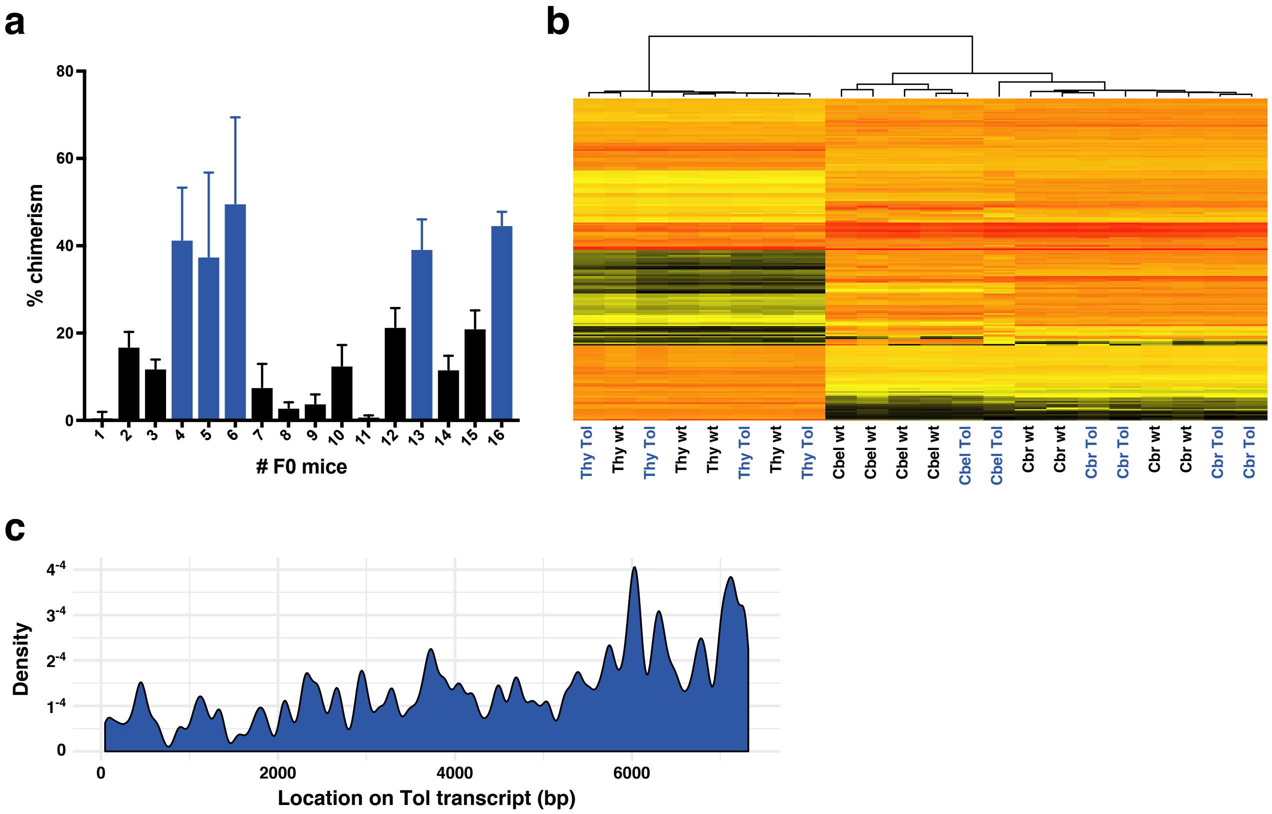


**Supplementary Fig. 2. Validation of the Tol mouse model. a**, Bar chart depicting the percentage of chimerism in F0 animals, as determined by quantitative PCR. Blue bars represent animals used for subsequent breeding. **b**, Hierarchical clustering analysis performed on RNA sequencing data from thymus, cerebrum and cerebellum of WT (black) and Tol (blue) mice. Complete-linkage clustering was performed on the top 1,000 most highly expressed genes in the dataset. Thy = thymus, Cbel = cerebellum, Cbr = cerebrum. **c**, Density of aligned reads along the *Tol* ORF. Data shown in (**b-c**) are representative of n=4 (Tol) and n=4 (WT) mice. PURO-R = puromycin resistance gene, TK = thymidine kinase gene, CPM = counts per million.

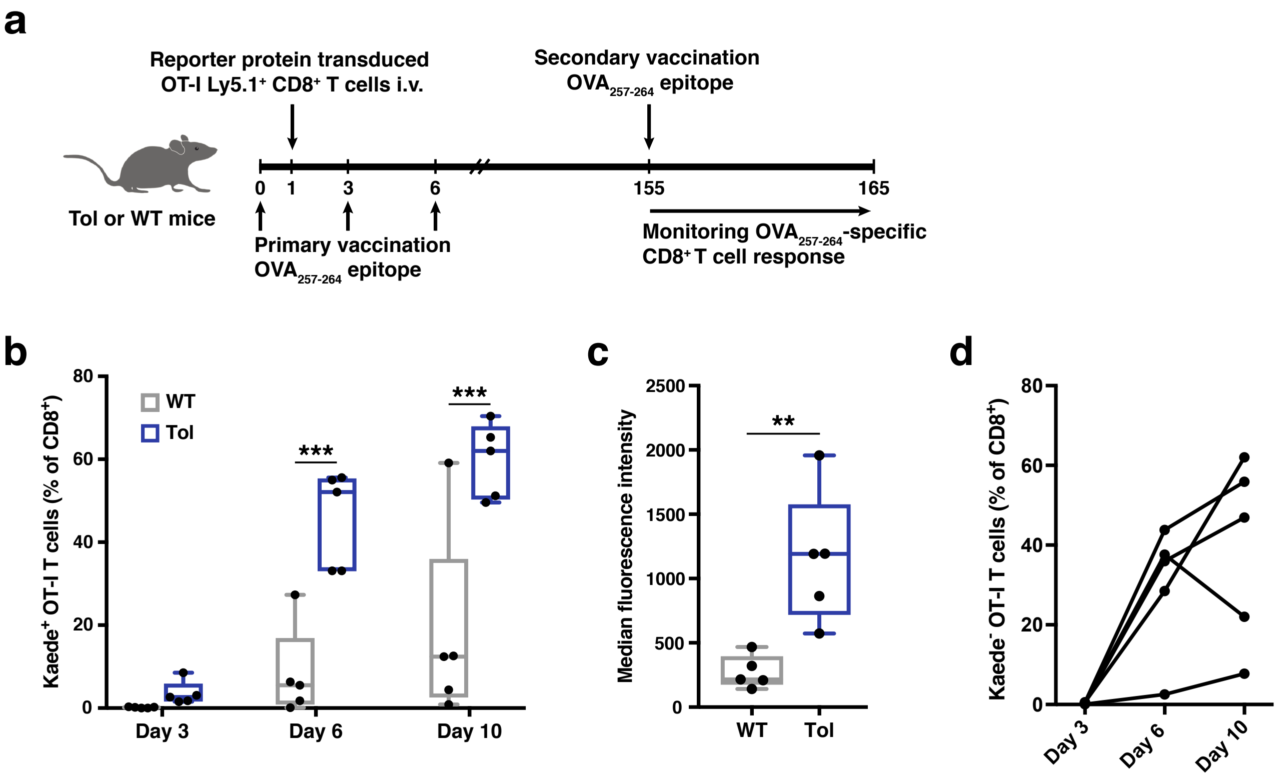


**Supplementary Fig. 3. Enhanced recall potential of Kaede+ OT-I T cells in Tol mice. a**, Schematic overview of adoptive cell transfer, vaccination and monitoring strategy. **b, c** Percentage and MFI of Kaede+ OT-I T cells after secondary vaccination. Note that both the number of Kaede+ OT-I cells and the Kaede expression level are increased in Tol mice relative to WT mice. **d,** Percentage of Kaede- OT-I T cells after secondary vaccination. Cells (**b-d**) are gated on IR-dye−CD8+ lymphocytes, and data depict n=5 (Tol) and n=5 (WT) mice. Box-plots (**b**, **c**) depict interquartile range, with whiskers representing minimum and maximum values. Dots (**b**, **c**) and lines (**d**) indicate individual mice. *P* values were determined by either Repeated Measures two-way ANOVA with Sidak's multiple comparisons test (**b**) or two-tailed Mann-Whitney signed rank test (**c**). $^{**}P < 0.01$; $^{***}P < 0.001$.

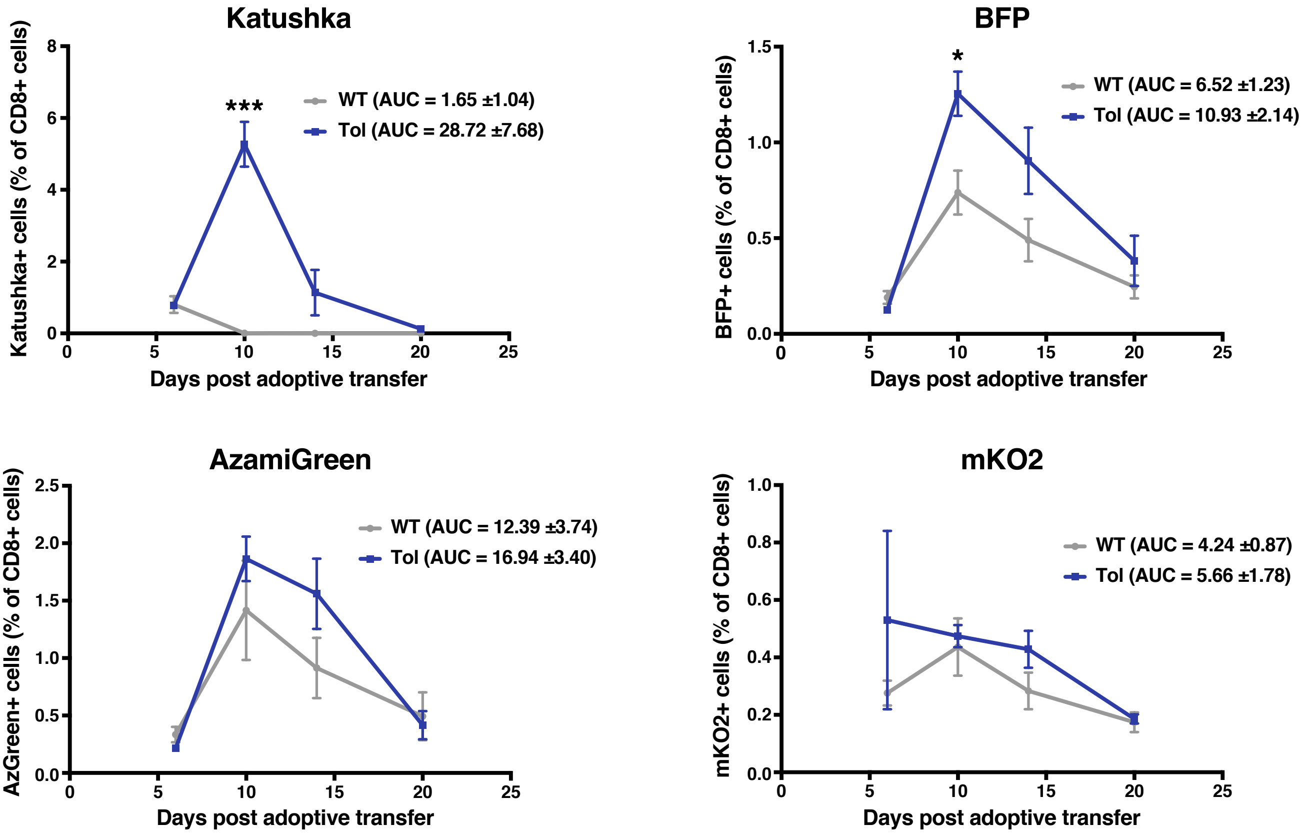


**Supplementary Fig. 4. Analysis of engraftment of Katushka, BFP, AzamiGreen and mKO2 modified T cells in WT and Tol mice.** Percentage $FP^{+}$ OT-I T cells detected in the blood of WT and Tol mice at indicated time-points. Lines represent group means of Tol (n=5) and WT (n=7) mice, error bars depict standard errors of the mean. Mean area under the curve (AUC) is given for each group. *P* values were determined by Repeated Measures two-way ANOVA with Sidak's multiple comparisons test to test the significance at each time point. *P < 0.05; **P < 0.01; ***P < 0.001.

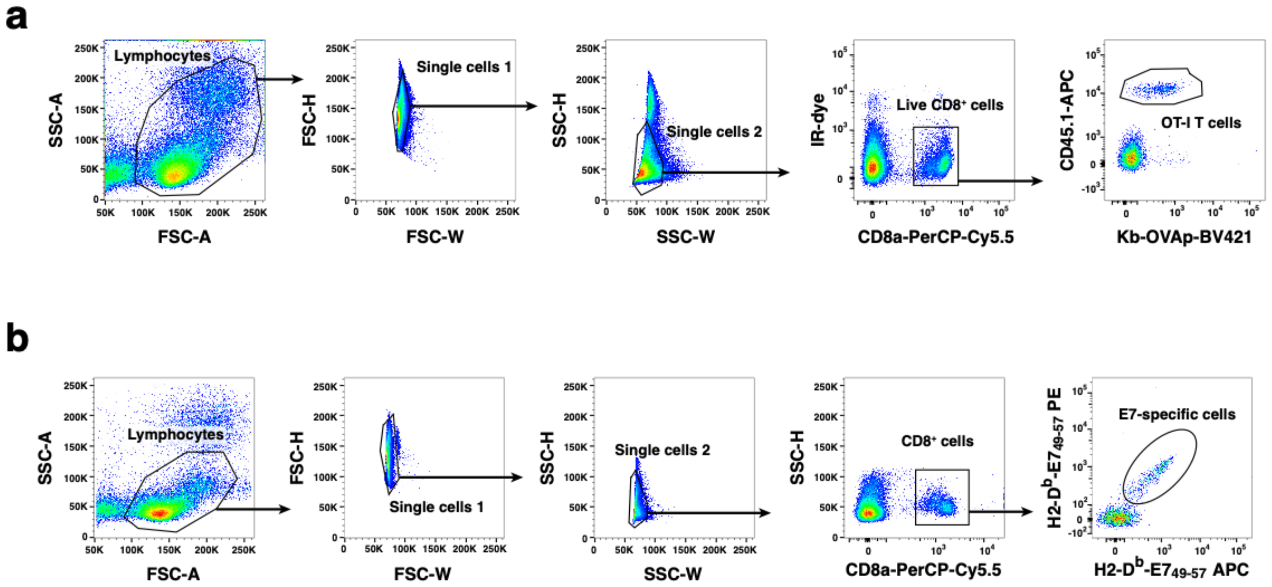


**Supplementary Fig. 5. Gating strategy for the detection of antigen-specific T cell responses in blood samples by flow cytometry. a**, Gating strategy applied to detect transferred OT-I T cell populations. **b**, Gating strategy applied to detect endogenous E7-specific T cell responses.

| Genotype | Sex | Histopathological analysis |
|---|---|---|
| **Tol** | F | Spleen: The lymphoid compartment showed a low cellular density in the marginal zone.<br>Thymus: Both cortex and medulla of the thymus showed hyperplastic changes with increased mitosis in the cortex. |
| **Tol** | F | Spleen: The lymphoid compartment showed a low cellular density in the marginal zone. Hematopoiesis in the red pulp was also low.<br>Liver: Focal necrotizing hepatitis.<br>Head: Ectopic thymus in the neck region.<br>Thymus: High mitotic rate in the thymic cortex. |
| **Tol** | F | Spleen: Increased mitotic rate in the lymphoid compartment, and low hematopoiesis in the red pulp of the spleen.<br>Thymus: High mitotic rate in the cortex of the thymus, accompanied by hypertrophy of the Hassall's bodies. |
| **Tol** | M | Skin: Focal and mild inflammatory lesions in the muscles of subcutis.<br>Thymus: Above average mitotic cells are present in the cortex. |
| **Tol** | M | Thymus: Above average mitotic cells are present in the cortex.<br>Spleen: Limited marginal zone around lymphoid compartment. |
| **Tol** | M | Thymus: Above average mitotic cells are present in the cortex.<br>Spleen: Very limited marginal zone around lymphoid compartment. |
| **WT** | F | Spleen: Low hematopoietic populations in the red pulp, and low cellular density in the marginal zone of the lymphoid compartment.<br>Liver: Mild and focal inflammatory infiltrations in the liver parenchyma.<br>Heart: Local lesions of myocarditis with lysis of the myocytes.<br>Thymus: Hyperplasia of the cortex with increased number of mitotic cells, accompanied by hypertrophy of the Hassall's bodies. |
| **WT** | F | Spleen: The lymphoid compartment showed a low cellular density in the marginal zone.<br>Thymus: Hyperplasia of the thymic cortex with increased mitotic cells, accompanied by hypertrophic changes of the Hassall's bodies. |
| **WT** | F | Pancreas: Degeneration/atrophy of the exocrine pancreatic parenchyma.<br>Thymus: High mitotic rate in the cortex of the thymus, accompanied by hypertrophy of the Hassall's bodies. |
| **WT** | M | Skin: Multiple local lesions of dermatitis, with extension to the subcutaneous muscles. |
| **WT** | M | Skin: Local lesions of myositis with necrosis of the muscles.<br>Muscles: Multiple local lesions of dermatitis, with extension to subcutaneous muscles. |
| **WT** | M | Thymus: Above average mitotic cells are present in the cortex.<br>Head: Small inflammatory lesions in the muscles of tongue with the presence of hair materials. Ectopic thymus in the neck region. |

**Supplementary Table 1**. Histopathological analysis from 7-week old Tol (blue) and WT (gray) male (M) and female (F) mice of skin, lymph node, thymus, testes, accessory sex glands, gastrointestinal tract, spleen, pancreas, kidney, liver, heart, lung, sternum and extremity, muscles and head. Only abnormalities are included in the table.
