# Supplementary material for: A mouse model that is immunologically tolerant to reporter and modifier proteins
Source: Commun Biol. 2020 May 29;3:273. doi: 10.1038/s42003-020-0979-0 (PMC7260180; doi:10.1038/s42003-020-0979-0)
Supplement: Supplementary file 2 — Description of Additional Supplementary Files [file 42003_2020_979_MOESM2_ESM.pdf]

Description of Additional Supplementary Files

File Name: **Supplementary Data 1**
Description: Potential H2-Db and H2-Kb ligands derived from the Tol ORF, calculated by NetMHCpan and NetChop. All peptides annotated as strong or weak binders (% rank > 2.0) are provided.

File Name: **Supplementary Data 2**
Description:
EdgeR 3.9 output for RNAseq analysis of cerebrum, cerebellum and thymus from Tol and WT animals.

File Name: **Supplementary Data 3**
Description: Source data underlying the graphs and charts presented in the figures.
