# Supplementary material for: A mouse model that is immunologically tolerant to reporter and modifier proteins
Source: Commun Biol. 2020 May 29;3:273. doi: 10.1038/s42003-020-0979-0 (PMC7260180; doi:10.1038/s42003-020-0979-0)
Supplement: Supplementary file 6 — Reporting Summary [file 42003_2020_979_MOESM6_ESM.pdf]

nature research

Corresponding author(s): T.N. Schumacher

Last updated by author(s): Apr 8, 2020

Reporting Summary

Nature Research wishes to improve the reproducibility of the work that we publish. This form provides structure for consistency and transparency in reporting. For further information on Nature Research policies, see Authors & Referees and the Editorial Policy Checklist.

Statistics

For all statistical analyses, confirm that the following items are present in the figure legend, table legend, main text, or Methods section.

n/a	Confirmed	
☐	☒	The exact sample size (*n*) for each experimental group/condition, given as a discrete number and unit of measurement
☐	☒	A statement on whether measurements were taken from distinct samples or whether the same sample was measured repeatedly
☐	☒	The statistical test(s) used AND whether they are one- or two-sided<br>*Only common tests should be described solely by name; describe more complex techniques in the Methods section.*
☒	☐	A description of all covariates tested
☒	☐	A description of any assumptions or corrections, such as tests of normality and adjustment for multiple comparisons
☐	☒	A full description of the statistical parameters including central tendency (e.g. means) or other basic estimates (e.g. regression coefficient) AND variation (e.g. standard deviation) or associated estimates of uncertainty (e.g. confidence intervals)
☐	☒	For null hypothesis testing, the test statistic (e.g. *F*, *t*, *r*) with confidence intervals, effect sizes, degrees of freedom and *P* value noted<br>*Give P values as exact values whenever suitable.*
☒	☐	For Bayesian analysis, information on the choice of priors and Markov chain Monte Carlo settings
☒	☐	For hierarchical and complex designs, identification of the appropriate level for tests and full reporting of outcomes
☒	☐	Estimates of effect sizes (e.g. Cohen's *d*, Pearson's *r*), indicating how they were calculated

Our web collection on statistics for biologists contains articles on many of the points above.

Software and code

Policy information about availability of computer code

Data collection	RNA single-end 65 bp sequencing was performed on a HiSeq 2500 System (Illumina). Flow cytometry data was collected on a Fortessa (BD Biosciences).
Data analysis	Differential gene expression analysis was performed using EdgeR (version 3.9). Flow cytometry data was analyzed in FlowJo (version 10.4.2).

For manuscripts utilizing custom algorithms or software that are central to the research but not yet described in published literature, software must be made available to editors/reviewers. We strongly encourage code deposition in a community repository (e.g. GitHub). See the Nature Research guidelines for submitting code & software for further information.

Data

Policy information about availability of data

All manuscripts must include a data availability statement. This statement should provide the following information, where applicable:

- Accession codes, unique identifiers, or web links for publicly available datasets
- A list of figures that have associated raw data
- A description of any restrictions on data availability

Mouse cDNA sequences (GRCm38) used for Salmon alignment were downloaded from Ensembl (http://www.ensembl.org/biomart/martview). Data supporting the findings of this study are available from the corresponding author upon reasonable request.

Field-specific reporting

Please select the one below that is the best fit for your research. If you are not sure, read the appropriate sections before making your selection.

☒ Life sciences ☐ Behavioural & social sciences ☐ Ecological, evolutionary & environmental sciences

For a reference copy of the document with all sections, see nature.com/documents/nr-reporting-summary-flat.pdf

Life sciences study design

All studies must disclose on these points even when the disclosure is negative.

Sample size	Sample sizes of experiments were chosen based on prior research conducted in our laboratories.
Data exclusions	No data in this study were excluded.
Replication	The number of experiments performed, in all cases supporting the data reported, are stated in the figure legends.
Randomization	Tol and WT mice were randomly assigned to experimental conditions.
Blinding	Histopathological sections were evaluated and scored by an animal pathologist blinded to animal genotype.

Reporting for specific materials, systems and methods

We require information from authors about some types of materials, experimental systems and methods used in many studies. Here, indicate whether each material, system or method listed is relevant to your study. If you are not sure if a list item applies to your research, read the appropriate section before selecting a response.

Materials & experimental systems

n/a	Involved in the study	
☐	☒	Antibodies
☐	☒	Eukaryotic cell lines
☒	☐	Palaeontology
☐	☒	Animals and other organisms
☒	☐	Human research participants
☒	☐	Clinical data

Methods

n/a	Involved in the study	
☒	☐	ChIP-seq
☐	☒	Flow cytometry
☒	☐	MRI-based neuroimaging

Antibodies

Antibodies used	- anti-CD45.1-APC (clone: A20, cat. #: 17-0453-81, company: Thermo Fisher Scientific, validated on: mouse splenocytes , dilution: 1:200)<br>- anti-CD8-PerCp-Cy5.5 (clone: eBioH35-17.2, cat. #: 25-0083-82, Thermo Fisher Scientific, dilution: validated on: mouse splenocytes, dilution: 1:200)<br>- E7-49-67-PE/APC and OVA-257-264-PE/BV421 multimers (produced in-house, see 'Methods-section 'Analysis of antigen-specific T cell responses by flow cytometry', dilution: 1:50)
Validation	Antibodies were validated for their application by the manufacturer (see above). Antigen-specific T cell populations were stained with two multimers of the same specificity labeled in a different color and only double-positive cells were included in the analyses. Multimer batches were validated on murine blood or spleen samples.

Eukaryotic cell lines

Policy information about cell lines

Cell line source(s)	Phoenix-Eco retro virus packaging cells obtained from ATCC (ATCC® CRL-3214™).
Authentication	Cell line was authenticated based on cellular morphology.
Mycoplasma contamination	Cell lines were tested negative for Mycoplasma contamination.
Commonly misidentified lines (See ICLAC register)	No commonly misidentified cell lines were used in this study.

Animals and other organisms

Policy information about studies involving animals; ARRIVE guidelines recommended for reporting animal research

Laboratory animals	For details on the 'Tol' transgenic mouse model, see Methods-section 'Generation of Tol transgenic mice'. C57BL/6-Ly5.1 and OT-I mice were obtained from Jackson Laboratories, crossed to obtain C57BL/6-Ly5.1-OT-I donor mice for adoptive transfer experiments and used for experimentation at 7-14 weeks old.
Wild animals	The study did not involve wild animals.
Field-collected samples	The study did not involve samples collected from the field.
Ethics oversight	All animal experiments were approved by the Animal Welfare Committee of the NKI, in accordance with national guidelines.

Note that full information on the approval of the study protocol must also be provided in the manuscript.

Flow Cytometry

Plots

Confirm that:

☒ The axis labels state the marker and fluorochrome used (e.g. CD4-FITC).

☒ The axis scales are clearly visible. Include numbers along axes only for bottom left plot of group (a 'group' is an analysis of identical markers).

☒ All plots are contour plots with outliers or pseudocolor plots.

☒ A numerical value for number of cells or percentage (with statistics) is provided.

Methodology

Sample preparation	Sample preparations are described in the Materials and Methods-section 'Analysis of antigen-specific T cell responses by flow cytometry'.
Instrument	Samples were measured on a Fortessa (BD Biosciences).
Software	Samples were analyzed in Flow Jo (version 10.4.2).
Cell population abundance	No sorts were performed for this study.
Gating strategy	Samples were gated on lymphocytes by FSC-A/SSC-A gating and doublets were subsequently gated out by FSC-A/FSC-W. Dead cells were excluded by gating on near-IR-negative cells, CD8+ T cell were then selected by gating on CD8-PeCP-Cy5.5 positivity and plots depict indicated populations. Gates were placed based on negative populations.

☒ Tick this box to confirm that a figure exemplifying the gating strategy is provided in the Supplementary Information.
